# Combination of early Interleukin-6 and -18 levels predicts postoperative nosocomial infection

**DOI:** 10.3389/fendo.2022.1019667

**Published:** 2022-10-10

**Authors:** Qingwei Yu, Chaoqun Cen, Min Gao, Hong Yuan, Jingjing Liu

**Affiliations:** ^1^ Clinical Research Center, The Third Xiangya Hospital, Central South University, Changsha, China; ^2^ Department of Emergency Medicine, Third Xiangya Hospital, Central South University, Changsha, China; ^3^ Department of Intensive Medicine, Third Xiangya Hospital, Central South University, Changsha, China

**Keywords:** abdominal surgery, postoperative nosocomial infection, early prediction, inflammatory factors, interleukin

## Abstract

**Background:**

The inflammatory response plays a critical role in postoperative nosocomial infections, which are the most common postoperative complications causing adverse events and poor postoperative outcomes. This study aimed to explore the ability of early inflammation-related factor levels to predict the occurrence of nosocomial infections after abdominal surgery.

**Methods:**

The study included 146 patients with open abdominal surgery (a nosocomial infection group (NI group, n=42) and a no-nosocomial infection group (NNI group, n=104)). After 1:1 matching, the patients were divided into a matching nosocomial infection group (M-NI group, n=25) and a matching no-nosocomial infection group (M-NNI group, n=25). Serum levels of interleukin (IL)-6, IL-8, IL-10, IL-12, IL-18, macrophage migration inhibitory factor (MIF), and monocyte chemotactic protein (MCP-1) were tested at three time points (pre-operation, 0-hour post-operation (POD1) and 24-hour post-operation (POD2)). The area under the receiver operating characteristic curve (AUC-ROC) was used to test the predictive abilities.

**Results:**

There were significant differences in the levels of IL-6, IL-12, and IL-18 between the M-NI and M-NNI groups (p < 0.05), but not in the levels of other inflammatory factors. MIF, IL-8, and MCP-1 levels were higher in the M-NI group than in the M-NNI group at POD2 (p < 0.05). In the ROC analysis, the AUC for prediction of nosocomial infection using a combination of IL-6 and IL-18 at POD1 was 0.9616, while the AUCs for IL-6 alone and IL-12 alone were 0.8584 and 0.8256, respectively.

**Conclusions:**

The combination of the levels of inflammatory factors, IL-6 and IL-18, at the 0-hour postoperative time point, significantly improved the predictive ability to the development of postoperative infection during perioperative period. Our study suggests the importance of monitoring postoperative inflammatory markers.

## Introduction

Postoperative nosocomial infection which is caused by the environment or the staff in the hospital, significantly prolongs hospitalization, increases the cost of hospitalization, and directly affects the mortality and disability rates of inpatients (1). Identifying biomarkers of nosocomial infection and performing interventions at the early stages is necessary to decrease death or disability. However, few studies have explored this area and provided effective predictors of postoperative nosocomial infection.

Previous studies have suggested that patients undergoing major surgery often present with host immunosuppression, which is caused by surgical stress, anesthesia, blood transfusion, and opioids (2–5). Immunosuppression impairs the patient’s internal defense mechanisms, leading to susceptibility to infection. The dysfunctional inflammatory response caused by surgical stress is an important mechanism of host immune injury. Although previous studies reported the involvement of some cytokines in this response, there is still much debate on this topic. It is difficult to use clinical indicators alone to evaluate inflammatory responses to surgical stress, suggesting that biomarkers of inflammation need to be combined.

Inflammatory chemokines, cytokines, free radical oxygen species, and injury-related molecular model molecules are the main regulatory factors of the body’s inflammatory response to surgical stress. Studies have shown that the degree and mode of the inflammatory cascade after stress injury may be related to the patient outcome. Recent studies have mostly focused on the predictive abilities of the neutrophil-to-lymphocyte ratio, monocyte-to-lymphocyte ratio, platelet-to-lymphocyte ratio, serum fibrinogen level, and C-reactive protein (CRP) level for the occurrence of post-operative inflammation (6–10). Few studies have explored the effect of combined interleukins (ILs), with only inconsistent results in the few reports (11, 12). Moreover, researchers have explored the effects of these potential predictive biomarkers on the pre-operative status, ignoring the possible effect on the early status after the operation.

Our study intends to identify specific, early postoperative, inflammatory factors among patients with nosocomial infections after abdominal surgery, to help establish an early evaluation tool to screen patients who are at high risk of inflammation.

## Materials and methods

### Patient enrollment and data collection

We included a prospective collection of patients who underwent open abdominal surgery at the Third Xiangya Hospital of Central South University from July 2017 to December 2017.

Patients eligible for enrollment in the study were at least 18 years of age, and had undergone open abdominal surgery at the Third Xiangya Hospital of Central South University from July 2017 to December 2017. Herein, open abdominal surgery refers to open transabdominal surgery, such as gastrointestinal surgery, hepatobiliary surgery, pancreatic operation, spleen surgery, appendix operation and abdominal vascular surgery. However, abdominal wall operation, hernia surgery, retroperitoneal surgery, and minimally invasive surgery are excluded from the definition of “open abdominal surgery”.

Ineligibility criteria were pregnancy, infectious diseases before surgery, transplantation, long-term use of immunosuppressants, and any blood system disease involving the granulocyte system. Additionally, we excluded patients with a postoperative stay of ≤ 48 hours, with an unconfirmed diagnosis of postoperative infection, or lacking any data.

Patients’ clinical details included basic information and the clinical information related to the perioperative period.

The study was approved by the Ethics Review Committee of The Third Xiangya Hospital, Central South University (No. R17008). Written informed consent was obtained from all participants.

### Study design

A total of 160 patients were enrolled in our study. After excluding eight patients for lack of information or sample collection, two patients for postoperative stays of ≤ 48 hours, and four patients for un-identified clinical outcomes, 146 cases were enrolled and divided into two groups: a nosocomial infection group (NI group) (n=42) and a no-nosocomial infection group (NNI group) (n=104). After 1:1 matching according to age, sex, surgery conditions, and preoperative comorbidities, the patients were assigned to a matching nosocomial infection group (M-NI group, n=25) and a matching no-nosocomial infection group (M-NNI group, n=25) to explore the differences in the inflammatory factors.

### Blood collection and analysis of inflammation biomarkers

Blood samples were collected into citrated tubes *via* venous or arterial catheters at three-time points (pre-operation (Prep), 0-hour post-operation (POD1), and 24-hour post-operation (POD2). The blood samples were centrifuged, and serum aliquots were stored in cryoprecipitate tubes at −80°C for the analysis of inflammatory mediators. The Luminex 100 IS (Luminex, Austin, TX) was used to measure the serum levels of IL-6, IL-8, IL-10, IL-12, IL-18, macrophage migration inhibitory factor (MIF), monocyte chemotactic protein (MCP-1). The Luminex system was used according to the manufacturer’s instructions.

### Clinical outcomes

Nosocomial infection refers to the infection acquired by inpatients, including the infection that occurs during hospitalization, and the infection that acquires in hospital but occurs after hospital discharge. It does not include infection that had started before admission or existed at the time of admission.

The incidence of nosocomial infection must be identified by the nosocomial surveillance of intrahospital infections or identified by a review committee comprising three specialist infectious diseases physicians.

### Statistical analysis

All data were analyzed using SPSS 21.0 and R software 3.4.4. Continuous variables were presented as mean ± standard deviation, while categorical variables were presented as frequency and percentage. Statistical differences were summarized using matched t-test, chi-square test, or rank-sum test, as deemed appropriate. P < 0.05 was considered statistically significant. Odds ratio (OR) was determined using paired logistic regression. The receiver operating characteristic (ROC) curve and area under the ROC curve (AUC) were used for predictions of nosocomial infection in three models (IL-6, IL-18, and a combination of IL-6 and IL-18). Sensitivity and specificity for the fitted values are shown in **Figure 3**.

## Results

### Demographics and outcomes

In this study, 146 patients were included, of whom 92 were male and 54 were female. Among the patients, there were 42 patients (69.0% male) in the NI group, accounting for 28.8% of all the patients, with an average age of 56.8 years. The NNI group had 104 patients (62.5% male), accounting for 71.2% of all the patients, with an average age of 56.2 years.

In the NI group, 12 patients had two or more nosocomial infections, including 3 patients with three or more infections. During their hospitalization, 42 patients in the NI group had 58 infections (1.3 episodes per patient) with an infectious event rate of 39.7%, of which 8 patients had multiple-site infections (19%). The sites of infections showed in the following decreasing order of frequency: lung, surgical site, urinary tract, and bloodstream infections.

After 1:1 matching according to age, sex, surgical classification (Level 3/4), surgery type (elective surgery), and operative method (open abdominal), the M-NI and the M-NNI groups were assigned. The characteristics of the patients in both groups are presented in **Table 1**. There was no statistically significant difference in several intraoperative conditions (intraoperative blood transfusion rate, intraoperative blood transfusion volume, intraoperative hypotension), laboratory metrics (preoperative and postoperative), and associated diseases (acute kidney injury [AKI] and chronic kidney disease [CKD]). Nevertheless, the operating time presented a statistical difference. In the M-NI group, five patients had two or more infections. Among all 25 patients, 17 patients had nosocomial infections of the lung, and eight patients had infections of surgical sites.

**Table 1:** Characteristics of patients in the M-NI group and M-NNI group.

Factors	Total number (n=50)	M-NI Group (n=25)	M-NNI Group (n=25)	P Value
**Demographic Data**				
Age	60 ± 14	60 ± 14	61 ± 13	0.643
Male, n (%)	34 (68.0)	17 (68.0)	17 (68.0)	–
**Intraoperative data**				
Operating time (min)	232 (161,296)	244 (208,299)	208 (131,287)	< 0.001
Minimally invasive, n (%)	0 (0)	0 (0)	0 (0)	–
Elective surgery, n (%)	50 (100.0)	25 (100.0)	25 (100.0)	–
Intraoperative blood transfusion rate, n (%)	24 (48)	14 (56.0)	10 (40.0)	0.26
Intraoperative blood transfusion volume (ml)	700 (400,1200)	800 (600,1200)	700 (400,1200)	0.23
Intraoperative hypotension, n (%)	16 (32.0)	11 (44.0)	5 (20.0)	0.069
**Laboratory Metrics**				
F-W (*10^9^)	7.05 ± 3.21	7.49 ± 3.60	6.62 ± 2.78	0.340
F-N (*10^9^)	5.06 ± 3.17	5.65 ± 3.66	4.47 ± 2.52	0.192
F-L (*10^9^)	1.35 ± 0.73	1.19 ± 0.51	1.51 ± 0.87	0.129
F-M (*10^9^)	0.42 ± 0.16	0.44 ± 0.13	0.40 ± 0.13	0.334
F-ALB (g/L)	38.89 ± 6.67	39.04 ± 7.80	38.75 ± 5.46	0.880
L-W (*10^9^)	14.54 ± 5.36	15.33 ± 5.62	13.75 ± 5.08	0.304
L-N (*10^9^)	12.89 ± 5.12	13.74 ± 5.44	12.03 ± 4.74	0.241
L-L (*10^9^)	0.81 ± 0.49	0.75 ± 0.54	0.88 ± 0.43	0.370
L-M (*10^9^)	0.89 ± 0.38	0.91 ± 0.39	0.88 ± 0.38	0.798
L-ALB (g/L)	29.49 ± 5.13	29.13 ± 5.49	29.87 ± 4.81	0.621
**Associated Diseases**				
CKD, n (%)	8 (16.0)	4 (16.0)	4 (16.0)	1
AKI, n (%)	8 (16.0)	5 (20.0)	3 (12.0)	0.440
**Infection sites**				
Surgical site, n (%)	8 (32.0)	8 (32.0)		
Lung, n (%)	17 (68.0)	17 (68.0)		
Urinary tract, n (%)	2 (8.0)	2 (8.0)		
Abdominal, n (%)	4 (16.0)	4 (16.0)		
Bloodstream, n (%)	1 (4.0)	1 (4.0)		

Continuous variables were presented as mean ± standard deviation, while categorical variables were presented as frequency and percentage.

AKI, acute kidney injury; CKD, chronic kidney disease; F-ALB, preoperative albumin level; F-L, preoperative lymphocyte count; F-M, preoperative monocyte count; F-N, preoperative nertrophil count; F-W, preoperative white blood cell count; L-L, postoperative lymphocyte count; L-M, postoperative monocyte count; L-N, postoperative nertrophil count; L-W, postoperative white blood cell count; M-NI group, matching nosocomial infection group; M-NNI group, matching no-nosocomial infection group.

### Circulating inflammatory factors and nosocomial infection

**Figure 1:**
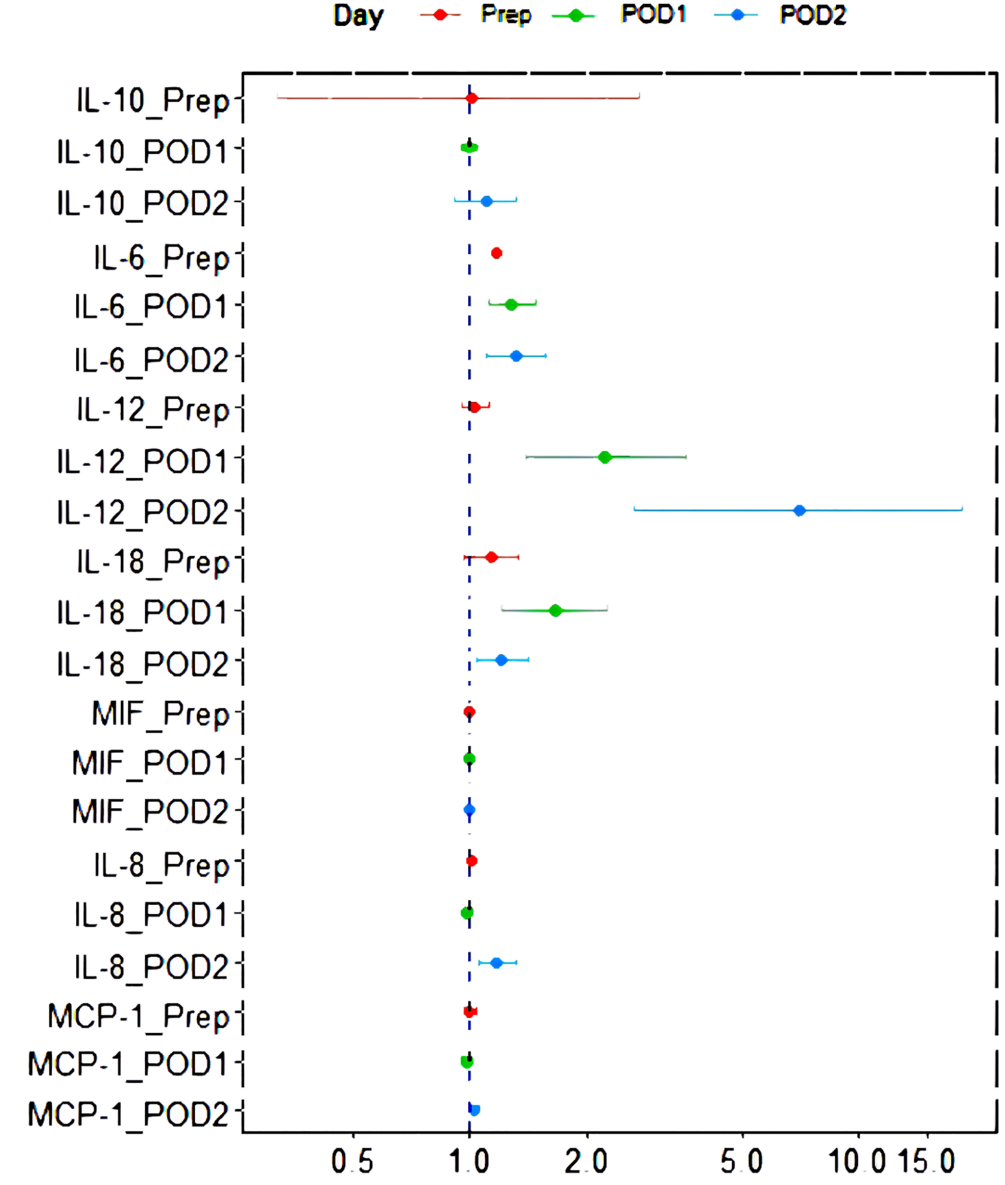
Forest plot of the relationship between perioperative inflammatory factor expression and postoperative infection outcome after the adjustment of operating time and low blood pressure in operation. IL, interleukin; MCP-1, monocyte chemoattractant protein; MIF, macrophage migration inhibitory factor; POD1, 0-hour post-operation; POD2, 24-hour post-operation; Prep, pre-operation.

The relationship between perioperative inflammatory factor expression and postoperative infection outcome is presented in **Figure 1**. After the adjustment of operating time and intraoperative hypotension, levels of IL-6 (Prep, POD1, POD2), IL-12 (POD1, POD2), IL-18 (POD1, POD2), and IL-8 (POD2) were found to be correlated with postoperative infection outcomes (p < 0.05).

**Figure 2:**
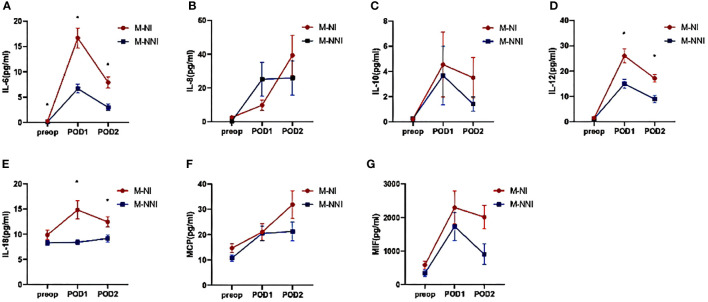
Serum inflammatory factor levels on pre-operation, 0-hour post-operation, and 24-hour post-operation. **(A)** The levels of IL-6 increased after the surgery and were significantly different between the M-NI and the M-NNI groups at three-time points. The time taken to reach the peak level was at POD1. **(B, C)** The increasing levels of IL-8 and IL-10 did not show significant differences between the M-NI and the M-NNI groups. **(D)** The levels of IL-12 were significantly different between M-NI and M-NNI groups at POD1 and POD2. The time to peak was at POD1. **(E)** The postoperative levels of IL-18 were significantly higher in the M-NI group and the time to peak in the M-NI group was POD1. **(F, G)** MCP and MIF showed higher levels in the M-NI group than in the M-NNI group at POD2. IL, interleukin; MCP, monocyte chemoattractant protein; MIF, macrophage migration inhibitory factor; M-NI, matching nosocomial infection group; M-NNI, matching no-nosocomial group; POD1, 0-hour post-operation; POD2, 24-hour post-operation; Preop, pre-operation.

**Figure 2** demonstrates the difference in circulating inflammatory factors between patients who developed NI and those who did not. The levels of IL-6, IL-12 and IL-18 were significantly different between the M-NI and the M-NNI groups (p < 0.05). The levels of the inflammatory factors (IL-6, IL-12, and IL-18) increased after the surgery, although the time taken to reach the peak levels were inconsistent. Meanwhile, several inflammatory factors such as MIF, IL-8, and MCP-1, showed higher levels in the M-NI group than in the M-NNI group at POD2 (p < 0.05).

### Prediction of postoperative nosocomial infection

**Figure 3:**
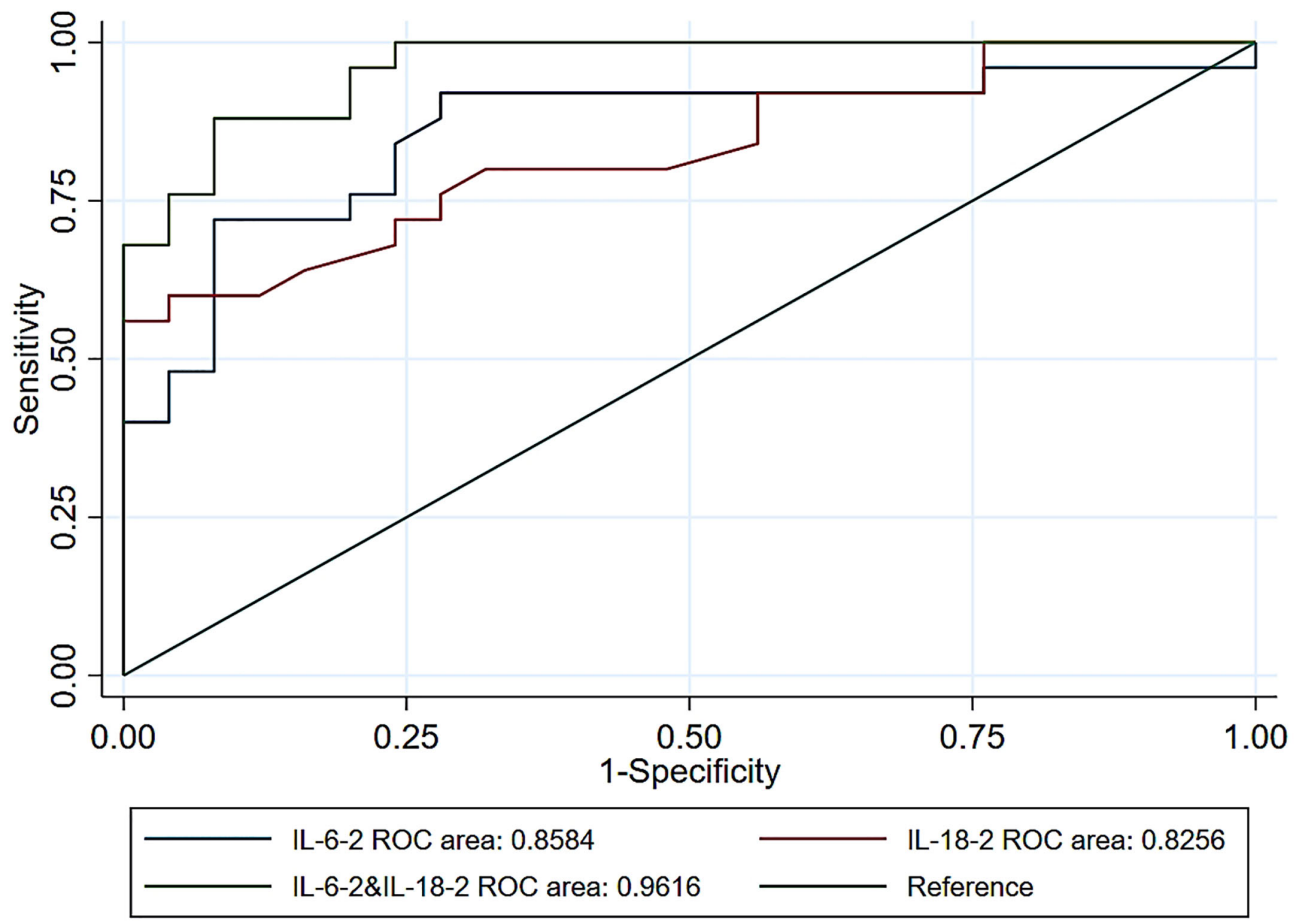
Receiver operating characteristic curves for postoperative infection prediction using IL-6 levels alone or IL-18 levels alone, and IL-6 combined with IL-18 levels at POD1. IL, interleukin; POD1, 0-hour post-operation; ROC, receiver operating characteristic.

**Figure 3** depicts the AUC for the univariable and multivariable associations of the inflammatory factors with the occurrence of postoperative infection at POD1. In the multivariable model, the highest OR of IL-6 levels for the occurrence of postoperative infection was found at POD2 (OR = 1.31, 95% confidence interval [CI] = 1.10-1.57, AUC = 0.8008). However, the highest OR of the IL-18 levels was found at POD1 (OR = 1.65, 95% CI =1.21-2.25, AUC = 0.8256). Based on the results of inflammatory factors in the multivariable model, the highest OR of 1.36 (95% CI = 1.13-1.65) was found for the combination of IL-6 and IL-18 at POD1, with a maximum AUC of 0.9616. This indicated that the combination of early IL-6 and IL-18 levels was a good predictor of postoperative nosocomial infection in abdominal surgery recipients.

## Discussion

In the current study, we used multiple serum inflammatory markers at three time points during the perioperative period to screen more sensitive markers for abdominal inflammation and prognosis of surgical patients. According to the comparisons, we found significant differences in IL-6, IL-12, and IL-18 levels between the M-NI and M-NNI groups, which might be associated with the occurrence of postoperative nosocomial infections. The combined use of IL-6 and IL-18 levels at 0 hours postoperatively significantly improved the prediction of nosocomial infection.

Previous studies have provided evidence that patients with open abdominal surgery are at high risk to contract nosocomial infections, with an incidence rate ranging from 7.3% to 27.20% (13–15). The incidence of nosocomial infections even exceeded 35% in a cohort of patients over 45 years of age who underwent gastrointestinal surgery (16). Studies have confirmed that the body will develop uncontrolled systemic inflammatory response syndrome (SIRS) in severe stress, which can cause multiple organ dysfunction and death of patients mainly in the early stage of stress (17, 18). With the development of medical technology, most patients can overcome the early stage of SIRS, but then enter the more complex stage of immunosuppression.

Postmortem examinations of patients with sepsis reveal an overactivated inflammatory response characterized by immunosuppression in the late stage, which can last for several days or even weeks (19, 20), leading to the susceptibility of the body to infection in the later stage. Infection by opportunistic pathogens occurs, and uncontrolled infections eventually lead to the death of patients (21, 22). Due to the influence of SIRS on the prognosis of disease, researchers have focused on studying therapeutic methods to modulate the inflammatory response. Many intervention methods have achieved ideal results in *in vitro* experiments, animal experiments, and even small-sample clinical studies. However, large-scale clinical trials have failed (23, 24), which may be related to the heterogeneity of patients.

At present, there are few methods to investigate inflammatory disorders and immune damage in patients. Once high-risk patients are identified in the early perioperative period by screening for the occurrence of inflammatory disorders and immune damage, clinicians should be urged to determine the target population for inflammatory and immunotherapy intervention. To our knowledge, the effect of CRP has been widely explored; higher levels of pre-operative CRP are associated with an increased risk of inflammation in patients with renal surgery, peritoneal surgery, urinary tract surgery, and some other types of surgery (6, 10, 25). As important inflammatory factors, the role of interleukins has also raised concerns but with inconsistent results. Similar to our results, a previous study on the risk factors of sepsis showed that pre-operative IL-6 levels could predict the incidence of sepsis (11). A recent study, which enrolled 19 patients with pancreaticoduodenectomy, showed that patients with post-operative inflammation had lower levels of IL-12p40, IFN-γ, IL-6, and IL-10, but a higher level of TNF-α (12), warranting further exploration.

Previous studies have revealed the effects of IL-6, IL-12, and IL-18 on the immune system. IL-6 derived from monocyte is a major cytokine in postoperative inflammatory stress. It activates acute reactive proteins such as CRP and procalcitonin (26, 27). Together with granulocyte and granulocyte-macrophage colony-stimulating factors, IL-6 can stimulate the primitive granulocyte progenitor cells in the bone marrow. In addition, IL-6 can activate anti-inflammatory pathways, providing negative feedback for the production of pro-inflammatory cytokines TNF-α and IL-1. Previous studies have suggested that high IL-6 levels are associated with postoperative complications for patients after major abdominal surgery (28). IL-12 is an important cellular immune activator that can initiate the Th1 response (29). IL-12 can promote the release of IFN-γ by natural killer (NK) cells and CD4^+^ T cells, thereby promoting the proliferation of various immune cells, especially cytotoxic T cells, B lymphocytes and NK cells (30). Many stress factors during the perioperative period can inhibit or regulate IL-12 production. Evidence suggests that surgical stress can inhibit IL-12 production in plasma (31), which is in agreement with our findings. IL-18, which acts synergistically with IL-12, plays a pathogenic role in all inflammatory diseases, either by promoting Th1- or Th2-related responses (32). Previous studies have shown that the production of IL-18 increases during stress and it promotes INF-γ production (33). Supplementation with exogenous IL-18 in patients undergoing surgery can improve the immunosuppressive status of patients after surgery (34). Thus, these three inflammatory factors (IL-6, IL-12, and IL-18) are involved in the activation of the inflammatory response and are elevated in the early stages of nosocomial infection.

Our study focused on the relationship between inflammatory factors and nosocomial infection, showing their changes during the perioperative period. The ROC curve further provided evidence that the combination of IL-6 and IL-18 could better predict the occurrence of postoperative nosocomial infection. Unlike previous research, which just probed the levels of pre-operative inflammatory factors, we focused on the early post-operative stage and have provided a more credible prediction model. We also examined multiple inflammatory factors and verified the predictive ability of ILs, which is different from previous studies.

Some limitations of our studies should be considered. As an exploratory study, the number of patients included is small, causing bias. Owing to the limitations in the inclusion criteria, especially the definition of abdominal surgery, the generalization to other surgery patients of the predictors found in this study needs further exploration. However, the combination of clinical indicators and early inflammation indicators is a promising research direction that must be further investigated. In the next study, we plan to explore a larger sample size to further clarify the relationship between inflammatory factors and infection outcomes, and establish valuable biomarkers to predict the occurrence of postoperative complications more accurately.

## Conclusions

We found that changes in the expression of the inflammatory factors IL-6, IL-12, and IL-18 in the early perioperative period may be associated with the occurrence of postoperative nosocomial infections, and that the combined use of inflammatory factors IL-6 and IL-18 at 0 hours postoperatively significantly improved the perioperative prediction of the development of postoperative infection.

## Data availability statement

The raw data supporting the conclusions of this article will be made available by the authors, without undue reservation.

## Ethics statement

The studies involving human participants were reviewed and approved by the Ethics Review Committee of The Third Xiangya Hospital, Central South University (No. R17008). The patients/participants provided their written informed consent to participate in this study.

## Author contributions

Concept and design: JL, and HY. Performed experiment: JL and QY. Acquisition, analysis, or interpretation of data: JL, QY, CC, and MG. Drafting of the manuscript: JL, QY, CC, and MG. Critical revision of the manuscript for important intellectual content: JL, and HY. Statistical analysis: JL, QY, CC, and MG. Administrative, technical, or material support: HY. Supervision: JL and HY. All authors contributed to the article and approved the submitted version.

## Conflict of interest

The authors declare that the research was conducted in the absence of any commercial or financial relationships that could be construed as a potential conflict of interest.

## Publisher’s note

All claims expressed in this article are solely those of the authors and do not necessarily represent those of their affiliated organizations, or those of the publisher, the editors and the reviewers. Any product that may be evaluated in this article, or claim that may be made by its manufacturer, is not guaranteed or endorsed by the publisher.
